# The Effect of Outdoor Aeroallergens on Asthma Hospitalizations in Children in North-Western Tuscany, Italy

**DOI:** 10.3390/ijerph19063586

**Published:** 2022-03-17

**Authors:** Maria Di Cicco, Ester Del Tufo, Salvatore Fasola, Serena Gracci, Maria Giovanna Marchi, Luca Fibbi, Giovanna Cilluffo, Giuliana Ferrante, Diego G. Peroni, Stefania La Grutta

**Affiliations:** 1 Department of Clinical and Experimental Medicine, University of Pisa, Via Roma n. 55, 56126 Pisa, Italy; ester90dt@gmail.com (E.D.T.); serena_gracci@live.it (S.G.); diego.peroni@unipi.it (D.G.P.); 2 Allergology Section, Pediatrics Unit, Pisa University Hospital, Via Roma n. 67, 56126 Pisa, Italy; 3 Institute of Translational Pharmacology, IFT, National Research Council, 90146 Palermo, Italy; salvatore.fasola@community.unipa.it (S.F.); stefania.lagrutta@cnr.it (S.L.G.); 4 Regional Agency for Environmental Protection of Tuscany (ARPAT), 50144 Florence, Italy; giovannamarchi59@gmail.com; 5 Laboratory for Meteorology and Environmental Modelling (LaMMA Consortium), 50019 Sesto Fiorentino, Italy; fibbi@lamma.toscana.it; 6 Institute of Bioeconomy, IBE, National Research Council, 50019 Sesto Fiorentino, Italy; 7 Department of Earth and Marine Sciences, University of Palermo, 90123 Palermo, Italy; giovanna.cilluffo@unipa.it; 8 Department of Surgical Sciences, Dentistry, Gynecology and Pediatrics, Pediatric Division, University of Verona, 37126 Verona, Italy; giuliana.ferrante@univr.it

**Keywords:** allergy, Alternaria alternata, asthma, climate change, pollen, sensitization to outdoor allergens

## Abstract

Few data are currently available on the effects of aeroallergens in triggering respiratory symptoms in children. To evaluate the potential effects of daily outdoor aeroallergens loads on childhood admissions, in this case-crossover study, we analyzed data from 85 children hospitalized at the University Hospital of Pisa, Italy, for asthma or asthma-like symptoms without respiratory infection, between 2010 and 2019. Data were linked to outdoor allergens, temperature, nitrogen dioxide, and relative humidity observed during the same period. A 10-grains/m^3^ increase in the total aeroallergen concentration was associated with an increased risk of admission at lag 0 (OR = 1.054, 95% CI: 1.011–1.098), with a smaller effect at lag 1 (OR = 1.037, 95% CI: 1.008–1.067) and lag 2 (OR = 1.021, 95% CI: 1.003–1.039). Trends to larger effects were observed in children with sensitization to one or more aeroallergens (OR = 1.085, 95% CI: 1.004–1.173 at lag 0), in males (OR = 1.069, 95% CI: 1.009–1.132 at lag 0) and in older children (OR = 1.065, 95% CI: 1.007–1.127 at lag 0). Our study shows an association between increased outdoor allergens loads and asthma or asthma-like symptoms in children up to at least two days prior to hospitalization, suggesting that tracking aeroallergen counts may be useful to improve the management of respiratory allergic diseases.

## 1. Introduction

In the last decades, the prevalence of allergic respiratory diseases has been increasing worldwide, both in adults and children, especially in industrialized countries. The SIDRIA-2 study has shown that in Italy, asthma currently affects 9.3% of children and 10.3% of adolescents, while allergic rhinitis affects 12.3% of children and 20.9% of adolescents [1]. Well-known potential triggers for asthma exacerbations are represented by environmental factors such as day-to-day variation in weather conditions and air pollution, as well as aeroallergen counts, especially in sensitized individuals [2]. Many studies have evaluated the effects of variations in environmental pollen loads (both for single taxa and total pollen counts) on asthma exacerbations or asthma-like symptoms in terms of outpatient or emergency room (ER) visits in children, reporting conflicting results, especially with regard to thresholds above which symptoms may develop, due to heterogeneity in study design, study subject selection criteria, outcomes, investigated aeroallergens along with variable local conditions, and flora determining actual pollen exposure [3,4,5]. Moreover, most of the available studies have been focused on the respiratory effects of daily grass pollen counts, while little is known regarding other pollens. As a matter of fact, in 2018, a systematic review on the association between pollen counts and asthma ER visits in children and adolescents was carried out including 14 studies, but a meta-analysis was feasible only for three studies investigating grass pollen, which showed a statistically significant increase in the percentage change in the mean number of asthma ER presentations for an increase in 10 grass pollen grains/m^3^ of exposure (1.88%, 95% CI = 0.94–2.82%). Age-stratified studies found the strongest associations in children aged 5–17 years old [6]. A more recent systematic review by the same research group found similar results when investigating childhood asthma exacerbations requiring hospitalization: in particular, the study selected 12 studies that fulfilled the inclusion criteria, of which five studies were included in the meta-analysis for grass pollen, showing that an increase of 10 grains/m^3^ of exposure was associated with a 3% increase in childhood asthma admission (OR = 1.03; 95% CI: 1.01–1.04) [7].

The role of airborne fungal spores in triggering asthma-like symptoms in childhood has been elucidated even less due to similar issues related to the heterogeneity of the available studies. However, some studies suggest a significant association between fungal spore exposure and asthma ER evaluations and/or hospitalizations in children and adolescents [8,9].

Italy belongs to the Mediterranean region, which is characterized by temperate climate with mild winters and dry summers, and moderate rainfall concentrated in autumn and spring, and where the most common plants causing respiratory allergies belong to the Graminaceae, Urticacae, Cupressaceae, and Oleaceae families, with variability in distribution, in the flowering periods and in the duration of pollen seasons [10]. Notably, only a few studies have been carried out in our country to evaluate the effects of aeroallergens on childhood asthma. Tosca et al. investigated emergency calls for asthma exacerbation in children aged from 0 to 17 years recorded from 2002 to 2011 in Genoa, showing that the number of calls did not significantly change during the study period and found significant relationships between emergency calls and pollen counts (mainly Parietaria) during spring [11], while Bono et al. showed that ER admissions for respiratory diseases in a children’s hospital in Turin increased by 0.7% (95% CI: 0.1–1.2%) one day after a 10 grains/m^3^ increase in aeroallergens (Corylaceae, Cupressaceae, Gramineae, Urticaceae, Ambrosia, Betula) [12]. Noteworthy, most of the available studies on the effects of outdoor aeroallergens on hospitalization in children have included patients whose symptoms may have been triggered by respiratory infections.

The aim of this single-center study conducted in Pisa, Italy, was to assess the potential effect of daily outdoor allergen loads, including pollen and fungal spores, on childhood admissions for asthma or asthma-like symptoms in the north-western Tuscany, Italy, excluding those likely to have been caused by respiratory infections, in the period between 2010 and 2019.

## 2. Materials and Methods

### 2.1. Study Design and Area

In this case-crossover study, asthma admissions from 2010 to 2019 in children (age range 0–17 years) were linked to environmental data on outdoor allergens, nitrogen dioxide (NO_2_), temperature, and relative humidity observed during the same period. Clinical data relevant to the study were analyzed and reported anonymously; thus, the ethical research committee approval was waived. Pisa is a small urban area (185 km^2^) located a few kilometers from the mouth of the Arno River (5 m above sea level, 43°43′ N 10°24′ E), in a flat area with the Pisan mountain to the east (maximum altitude 917 m). It is characterized by residential areas and urban and inter-urban roads, and the average resident population in 2010–2019 consisted of approximately 88.800 individuals, according to data provided by ISTAT (the Italian national statistical institute, www.istat.it, accessed on 3 March 2022). According to data from the Laboratory for Meteorology and Environmental Modelling LaMMA Consortium (www.lamma.toscana.it, accessed on 3 March 2022), a public consortium between the Tuscany Region and the Italian National Research Council, on average, the warmest months in Pisa are July and August (average maximum air temperature 32.1 °C and 31.9 °C, respectively) and the coldest month is January (average minimum air temperature 1.8 °C). October and November are the wettest months; the average amount of annual precipitation is about 355 inches (900 mm), and the average annual percentage of humidity is about 72%, with December being the most humid month. The annual mean of wind speed in Pisa is about 11 km/h, corresponding to 2 in the Beaufort scale (light breeze).

### 2.2. Asthma-like Symptoms Admissions

Daily data on hospital admissions for asthma-like symptoms were retrieved from the medical records of patients aged < 18 years hospitalized at the Paediatrics Unit, University Hospital of Pisa, from January 2010 to December 2019. All the hospital admissions with asthma-like symptoms as the primary cause (International Classification of Diseases, 9th edition—ICD-9: 466.0, 493, 519.11 and 518.81) were considered in this study, while patients with a primary or secondary diagnosis of fever, upper and/or lower respiratory infection (suspected or confirmed by nasal swabs testing, blood examination, chest auscultation or chest X-rays), chronic respiratory diseases such as cystic fibrosis, primary ciliary dyskinesia, and bronchiectasis or foreign body inhalation or other distinct wheezing causes were excluded.

Each record included information about the date of hospital admission, primary diagnosis (ICD-9 code), age (categorized as 0–4 or 5–17 years), and gender. Atopy was defined based on the clinical history and on allergy tests performed during admission. Indoor sensitization was defined as positive skin prick test (wheal diameter ≥ 3 mm) or IgE antibody levels ≥ 0.35 kUI/L against house dust mites, cat or dog, while investigated pollen sensitization included Graminaceae (including Lolium perenne, Cynodon Dactylon, Phleum pratens), Urticaceae (Parietaria officinalis), trees (Olive, Cupressus, Pinus, Betula) and Ambrosia artemisifolia, which are the most important taxa causing allergic symptoms among people living in Tuscany. Data on sensitization to Alternaria alternata were also collected.

### 2.3. Outdoor Aeroallergen Counts

Daily mean outdoor aeroallergen counts (grains/m^3^) in the study area during the study period were retrieved from the Regional Agency for Environmental Protection of Tuscany (ARPAT) database according to the standard methods of the Aerobiology Italian Association (AIA). In Italy, a national aeroallergen surveillance system called POLLnet (www.pollnet.it, accessed on 3 March 2022) has been available since 2000, which coordinates 62 monitoring stations distributed in each of the 20 Italian regions; 4 stations are located in Tuscany, equipped with a seven-day Lanzoni VPPS 2000 spore trap (a Hirst volumetric sampler). The Lido di Camaiore station (34 m a.s.l.; 43°54′00″ N; 10°13′00″ E) is the closest to Pisa municipality area’s borders (about 15 km as the crow flies) and was chosen as the source of data for our study; the spore trap is situated on the flat roof of an 18-meter-high building, away from sources of pollution and with atmospheric circulation not affected by surrounding obstacles. Data on the following outdoor aeroallergens were collected: Ambrosia artemisifolia, Betula, Cupressus, Graminaceae, Olive, Pinaceae, Urticaceae. Data on Alternaria alternata spore concentration were also collected, considering its allergenic potential and widespread diffusion in Italy. Total daily concentrations were derived through the sum of all these aeroallergens.

### 2.4. Time-Varying Confounders

Summer periods of population decrease were defined as a categorical variable assuming value “1” from mid-July to end-August and “0” otherwise. Holidays were coded as a categorical variable assuming value “1” at Christmas and Easter (and the days around) and other isolated national holidays, and “0” during normal days. Influenza epidemic data were retrieved from the national Influenza surveillance system (InfluNet, www.iss.it, accessed on 3 March 2022) and were coded as a categorical variable assuming value “1” in the periods with the highest incidence and “0” otherwise. Daily means of air temperature and relative humidity in Lido di Camaiore during the study period were retrieved from LaMMA Consortium database. Dew point temperature was calculated from relative humidity [13]; an indicator of apparent temperature was calculated as a combination of air temperature and dew-point temperature [14]. Daily means of NO_2_ during the study period were retrieved from the ARPAT database, considering the Pisan background monitoring station of Passi (43°44′16″ N; 10°24′02″ E).

### 2.5. Statistical Analyses

An explorative analysis of asthma admissions, stratified by age group, was performed through frequency distributions of ICD-9 code, gender, and allergic sensitization. Group comparisons were carried out using Fisher’s exact test.

The association between outdoor aeroallergen counts and the risk of asthma admission was investigated using a case-crossover design [15]. This design is appropriate when the acute events (cases) are infrequent, and it can be regarded as a special type of case-control study. Specifically, a symmetrical and bidirectional selection of control days was performed to control for time trends and time-invariant covariates: the 7th day before the episode (day − 7) and the 7th day after (day + 7) [16]. Mean (standard deviation, SD) outdoor aeroallergen counts were compared between the case days (day 0) and the control days (day − 7 and day + 7) by testing for the relevant effect in a two-way ANOVA accounting for the matched data structure.

The annual means of total outdoor aeroallergen counts, apparent temperatures, and NO_2_ during the study period were visually displayed; linear trends were tested through linear regression. We also displayed the whole series of daily total outdoor aeroallergen counts, with bars superimposed to highlight the case days.

Outdoor aeroallergen effects were estimated through conditional logistic regression and were expressed as odds ratios (ORs) per 10 grains/m^3^ increase and 95% confidence intervals (CI). A preliminary power analysis was carried out for each aeroallergen based on the number of matched sets (the number of observed cases), the standard deviation of aeroallergen counts within the matched sets (calculated through a one-way ANOVA), and a range of expected ORs from 1.01 to 1.06 [17]. Since only total outdoor aeroallergens reached a power of 80% in the OR range (approximately at OR = 1.05; see Figure S1), we only included total aeroallergens in the subsequent analyses. The results for each pollutant are reported in the Supplementary Materials.

Lagged effects were estimated up to the 5th day before the episode. Due to the high collinearity of between-day aeroallergen counts, the lagged effects were constrained through linear functions [18]: a linear function in the lag and a linear function in the lag logarithm. The best lag structure was selected based on the Bayesian Information Criterion (BIC) (the lower, the better) [19]. All the effects were adjusted for summer population decreases, holidays, influenza epidemics, and apparent temperature. The effect of temperature was controlled for by calculating the average of current- and previous-day apparent temperature (lag 0–1) and using linear and quadratic terms [20]. The effect of NO_2_ was controlled for using linear terms (lag 0–1). However, an exploratory analysis showed that no effects of the aforementioned confounders were statistically significant, and the adjusted models had rather worse BICs compared to unadjusted models. Therefore, in order to obtain parsimonious models and improve the stability of the estimated effects, the confounders were not included in the final analyses [18].

Stratified analyses were performed by gender, age, and presence of sensitization to outdoor aeroallergens. A formal test of effect modification was performed for each risk factor (e.g., males vs. females) by setting up a unique model with two interaction terms. The first interaction term accounted for the change in the intercept of the linear lag function, while the second one accounted for the change in the slope. The significance of the interactions was assessed through a likelihood-ratio test comparing the full model (with interactions) and the reduced model (without interactions).

The following sensitivity analyses were run: excluding children under the age of 2 years; excluding cases that occurred before the end of March (early pollen season); using a time-stratified design for the selection of control days (the same days of the week within each month); adjusted by temperature; adjusted by NO_2_.

All the statistical analyses were performed through R version 4.0.2 (R Foundation for Statistical Computing, Vienna, Austria). Statistical significance was set at *p* < 0.05.

## 3. Results

### 3.1. Characteristics of the Study Population

A total of 85 asthma or asthma-like symptoms admissions occurred during the study period (Table 1). The mean age of the admitted children was 6.5 years (range: 0–17; SD = 4.5), 36 (42%) of whom were females, 40 (47%) were from 0 to 4 years old, and 35 (43%) had an outdoor sensitization. Moreover, 30 (35%) patients had a history of asthma and allergic rhinitis, 7 (8%) had a history of asthma without allergic rhinitis, and 28 (33%) had a history of allergic rhinitis without asthma; no child was admitted to an intensive care unit, but two patients (2.3%) developed pneumomediastinum and were treated with oxygen administration only. Acute bronchitis symptoms, meaning wheezing associated with generic lower respiratory tract inflammation, were more frequent in younger children, while asthma attacks were more frequent in older children. Older children were more frequently sensitized to aeroallergens (both indoor and outdoor).

### 3.2. Outdoor Aeroallergen Effects

Mean outdoor aeroallergen counts (grains/m^3^) on the case days were higher than on the control days, except for Betula and Graminaceae, even though differences were not statistically significant in most cases. Significant differences were found for Pinaceae and the total concentration (Table 2). Statistical power reached 80% only for the total outdoor allergen count (Figure S1).

**Figure 1:**
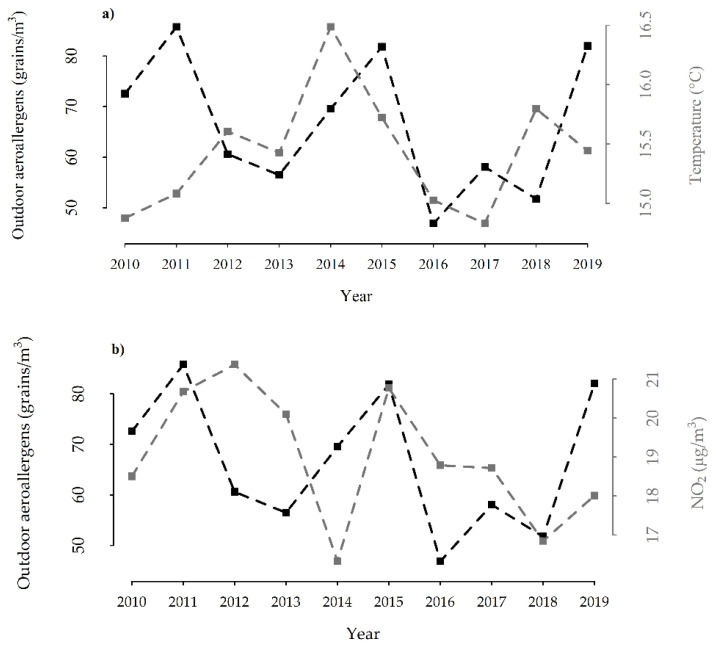
(**a**) Annual means of total outdoor allergen counts and apparent temperatures recorded in the study area; (**b**) annual means of total outdoor allergen counts and NO_2_.

**Figure 2:**
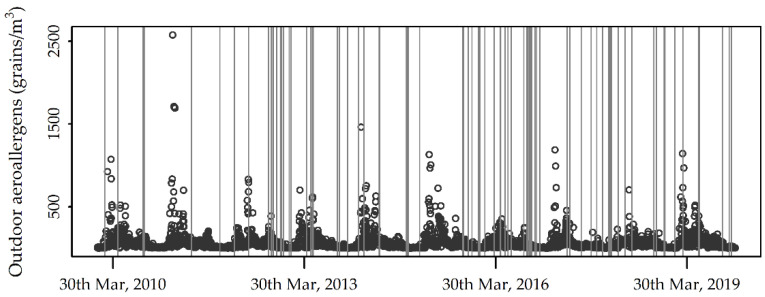
Daily total outdoor allergen counts recorded during the study period (points), with bars superimposed to highlight the case days.

The annual means of total outdoor allergen counts and apparent temperatures exhibited a similar trend starting from 2012 (Figure 1a), while the annual means of NO_2_ exhibited a somewhat different trend (Figure 1b). For all the variables, linear trends were not statistically significant. Figure 2 displays the whole series of daily total outdoor allergen counts, with bars superimposed to highlight the case days.

Based on the BIC, unadjusted models with a linear function in the lag yielded the best trade-off between goodness-of-fit and model parsimony. A 10-grains/m^3^ increase in the total outdoor aeroallergen concentration was significantly associated with an increased risk of asthma or asthma-like symptom admission at lag 0 (OR = 1.054, 95% CI: 1.011–1.098), with a smaller effect at lag 1 (OR = 1.037, 95% CI: 1.008–1.067) and lag 2 (OR = 1.021, 95% CI: 1.003–1.039) (Table 3). Concerning single aeroallergens, significant effects were only observed for Alternaria (OR = 1.140, 95% CI: 1.020, 1.275 at lag 0) (Table S1).

**Figure 3:**
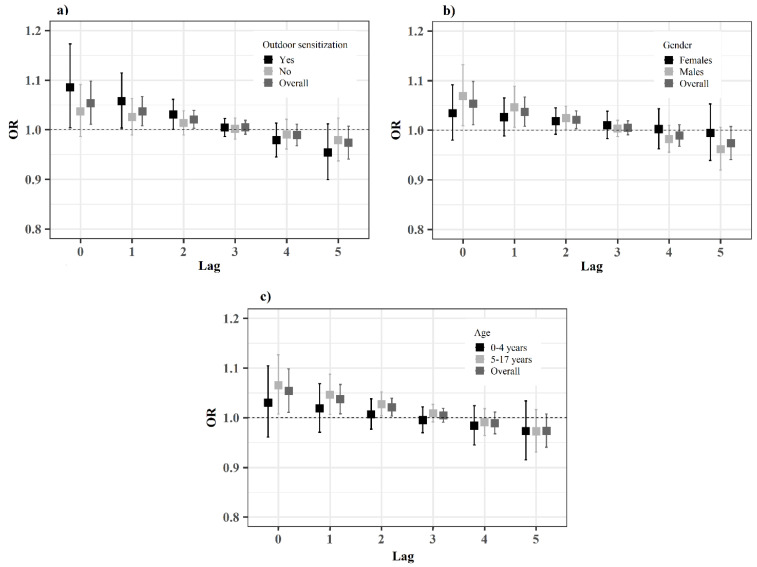
Acute effects of outdoor aeroallergen counts on the risk of asthma admissions: odds ratios (OR) per 10-grains/m^3^ increase and 95% confidence intervals for the conditional logistic regression models with distributed lags, stratified by presence of outdoor sensitization (**a**), gender (**b**), and age group (**c**).

In stratified models, we found trends to larger effects of outdoor allergen concentrations in children with sensitization to one or more aeroallergens (OR = 1.085, 95% CI: 1.004–1.173 at lag 0), in males (OR = 1.069, 95% CI: 1.009–1.132 at lag 0) and in older children (OR = 1.065, 95% CI: 1.007–1.127 at lag 0). However, no interaction effects were statistically significant (Table 3, Figure 3). Similar results were found in all the sensitivity analyses (Tables S2–S6).

## 4. Discussion

The current study shows that short-term exposure to increased total daily outdoor aeroallergen counts is associated with an increased risk of admission in children for asthma or asthma-like symptoms, with a stronger association in the case day and a lower association up to 2 days before hospitalization, with trends to larger effects in children with allergic sensitization, in males, and in children older than 5 years. Some previous studies have evaluated the effects of environmental pollens on exacerbations of respiratory symptoms in children, but most of them were focused on grass pollen and did not exclude children in whom exacerbation was triggered by respiratory infections. While these studies generally support the role of pollen exposure on both ER presentation and hospitalization in children [7,21,22], there is no consensus on the relative risks from different taxa or duration of exposure or threshold levels above which symptoms may occur [23]. In this scenario, our study adds evidence on the role of increased aeroallergen daily count as a trigger for asthma and asthma-like symptoms in children and adolescents.

Previously, the systematic review by Erbas et al. found strong associations between grass pollen exposure and asthma ER presentations in children older than 5 years [6]: considering that in our study, children aged ≥ 5 years were more frequently sensitized to both indoor and outdoor aeroallergens, we could infer that older children are more likely to suffer from allergic asthma and related exacerbations caused by increased aeroallergen loads, while preschoolers usually wheeze due to many different causes, including viral infections. It has also been reported that infants tend to be more sensitized to indoor allergens rather than to outdoor allergens [24,25]. However, we must acknowledge that although we have tried to exclude children with signs of acute respiratory infections, it may be possible that some of them escaped us.

Notably, in a recent study, Lee et al. analyzed the short-term effects of four groups of outdoor environmental factors (air pollutants, weather conditions, aeroallergens, and respiratory viral epidemics) on more than 28.000 asthma exacerbations that occurred in age-stratified groups (infants, preschool children, school-aged children, adults, and the elderly), among the total population in Seoul (Korea) from 2008 and 2012. The study results showed that pollen had significant effects in school-aged children and adults but not in younger children, while air pollution had significant short-term effects in all age groups [26]. A recent study has confirmed the role of pollutant concentrations in triggering childhood asthma exacerbations [27]. In our study, the effect of NO_2_ was not statistically significant, probably due to low statistical power.

With respect to the effect of single taxa, we found statistically significant associations only for Pinaceae (Table 2) and Alternaria (Table S1), consistently with the generally low statistical power highlighted in Figure S1. However, these significant results might simply be ascribed to a larger standard deviation of the associated daily counts (meaning a high variation in respective aeroallergen counts), yielding a slightly increased statistical power (especially for Pinaceae). Mean aeroallergen counts on the case days were not higher than on the control days for Betula and Graminaceae: indeed, pollinosis due to these taxa is less common than that caused by Parietaria and Olea in the Mediterranean area [28]. It should also be considered that thresholds for pollen counts above which symptoms occur may vary not only on the basis of the pollen type, loads, and sensitization but also depend on other exposures, including local weather conditions and air pollution. Nonetheless, it should be noted that in the literature, there is heterogeneity regarding the effect of single taxa, mostly related to the country where each study was carried out (as an example, significant effect for grass pollen in Spain and for tree pollen in the US) [22,29,30]; therefore, the inter-regional geographic and climatic influences may play a pivotal role in determining health consequences of aeroallergen exposure [31]. Given the well-known role of the investigated aeroallergens in causing respiratory symptoms [32], the aforementioned results should be evaluated, bearing in mind that it is not possible to evaluate whether each participant was exposed to the same levels of aeroallergens counted at a single site. Moreover, data on indoor aeroallergen exposure were not available for our study population.

While a stronger association between aeroallergen concentrations and hospitalization was expected in children with allergen sensitization, the stronger association in males is not easy to interpret and has been reported only in a few studies, such as a recent one showing higher odds of high asthma periods associated with grass pollen and some fungal spores for boys compared to girls [33]. Another study examining the contribution of outdoor fungi spores to child asthma admission reported that boys under 13 years and girls older than 15 years were more vulnerable [34], while girls appeared to be more vulnerable in a more recent case-crossover analysis [8]. Even if such an inconsistency in findings deserves to be further investigated, the potential higher male vulnerability may be linked to anatomical and functional differences between the developing airways in males and females and is in agreement with the higher prevalence of asthma in the male child population in Italy [1]. Boys have a higher number of alveoli and larger alveolar surface area than girls, but large airways tend to grow more slowly than parenchymal tissue in males, a phenomenon known as dysanaptic growth, with the result that they have relatively narrower airways. Such differences may be caused by the influence of sex hormones on lung development throughout childhood [35].

Our results are of particular interest when considering that an increase in pollen season duration and pollen production is expected in the next decades as a consequence of climate change [36,37], with detrimental effects on respiratory allergic diseases, especially in children, who are particularly vulnerable since they inhale a higher volume of air per body weight than adults and have an evolving respiratory and immune system [38]. As a matter of fact, warmer temperatures favor the growth of many plants, increase the number of allergenic proteins contained in pollen grains, increase pollen production itself, and anticipate and lengthen the pollen seasons [39]. In addition, the increase in CO_2_ due to fuel combustion leads to greater plant growth and an increase in pollen production, as shown in the case of Ambrosia [40,41]. Regarding molds, floods caused by increasingly frequent extreme weather events favor their growth [42]. As a consequence, patients affected by allergic rhinitis and asthma are expected to show increased severity and duration of symptoms, and a global increase in sensitization is also expected [36]. Even though we did not find a significant change in mean temperature and pollen concentrations during the whole study period, it should be noted that 10 years is a relatively short period to evaluate the effects of climate change. As a matter of fact, in studies evaluating longer periods, evidence emerged on significant changes already occurring in our country. As an example, in Bordighera, near Genoa, in the period between 1981 and 2007, a progressive increase in the duration of the pollen seasons for Parietaria, olive, and cypress was found, as well as an increase in their total pollen loads and in the percentages of patients sensitized to these pollens [43] Moreover, in the urban area of Genoa, an increase in Betulaceae, Urticaceae, Gramineae, and Oleaceae pollen counts, as well as Betulaceae sensitization, was reported between 1981 and 2010 [44].

One of the strengths of the current study is the case-crossover approach with a bidirectional selection of control days, which allowed us to adjust “by design” for time trends (day of the week, long-term and seasonal trends) and slowly varying or time-invariant factors (age, sex, aeroallergen sensitization). Another strength is the presence of several sensitivity analyses. Nonetheless, our study has several limitations. Firstly, the number of cases recorded in our study population was quite low; therefore, larger studies are needed to confirm our results. However, it should be noted that our study population is small mainly due to the fact that we have excluded every child with a history and/or clinical or laboratory signs of respiratory infection and/or chronic lung diseases, which is a strength in our study when considering that previous studies on this subject did not select children in such a way, but just speculated on the fact that autumn exacerbations are probably caused by viral infections spreading among children after school reopening. Secondly, we cannot firmly state that the association between increased aeroallergen counts and respiratory symptoms reflects a cause–effect relationship, considering that other triggers may be involved: unfortunately, we have not included weather conditions in our study, which are known to influence air quality in many different ways, such as pollen dispersion and long-distance transport determined by winds. Nevertheless, it should be noted that north-western Tuscany is not characterized by strong winds, especially during pollen season, so in our area, the role of wind in determining aeroallergens loads may have been limited to single days with bad weather conditions, especially in fall and winter. Even in this case, we might have caught variations considering the availability of daily data for single taxa concentrations during the study period. We also did not include levels of pollutants other than NO_2_ and patients and family habits (time spent outdoor vs. indoor). Data on indoor fungal spores, house dust mite levels, and tobacco smoke exposure were lacking too, but such elements should not determine short-term effects correlated with outdoor levels of aeroallergens.

Finally, only a single rooftop spore trap was used in this study, which was not located in the Pisa municipality area. However, Lido di Camaiore is just about 15 km away, and these locations share the height above the sea, weather conditions, and flora, with the result that we have a rough but reliable approximation of the average pollen exposure at ground level across the study area.

## 5. Conclusions

The current results report an association between increased environmental outdoor allergens loads and asthma or asthma-like symptoms in children. In particular, our data show a significant association up to at least two days prior to hospitalization, suggesting that tracking aeroallergen counts may be useful, especially for improving the management of children affected by respiratory allergic diseases. As an example, monitoring their levels through pollen calendars and smart devices may help people better plan their outdoor activities, including practicing physical exercise, limiting them when loads are too high to prevent the onset of respiratory symptoms. Moreover, pediatricians should improve their knowledge about the local environmental airborne allergens and vegetation in order to advise their patients accordingly.

## Figures and Tables

**Table 1:** Distribution of the 85 admissions for asthma-like symptoms by age group. Significant effects are in bold.

Study Population	Overall *n* = 85 (100%)	Age 0–4 Years *n* = 40 (47%)	Age 5–17 Years *n* = 45 (53%)	*p*-Value
Disease group (ICD-9 code)				**<0.001**
Acute bronchitis (466.0)	30 (35)	26 (65)	4 (9)	
Asthma attacks (493, 519.11)	46 (54)	9 (23)	37 (82)	
Acute respiratory failure (518.81)	9 (11)	5 (12)	4 (9)	
Gender				1.000
Female	36 (42)	17 (43)	19 (42)	
Male	49 (58)	23 (57)	26 (58)	
Aeroallergen sensitization ^§^				**<0.001**
No sensitization	24 (29)	23 (59)	1 (2)	
Indoor sensitization	23 (28)	9 (23)	14 (33)	
Outdoor sensitization	3 (4)	0 (0)	3 (7)	
Indoor and outdoor sensitization	32 (39)	7 (18)	25 (58)	
History of respiratory symptoms				**<0.001**
Asthma only	7 (8)	1 (3)	6 (13)	
Allergic rhinitis only	28 (33)	17 (42)	11 (25)	
Asthma and allergic rhinitis	30 (35)	2 (5)	28 (62)	
None	20 (24)	20 (50)	0 (0)	

Data are reported as No. (%). ^§^ Unavailable for 3 children.

**Table 2:** Mean (SD) outdoor aeroallergen counts (grains/m^3^) on the case days (day 0) and on the control days (day − 7 and day + 7). *p*-values are from two-way ANOVA. Significant effects are in bold.

Outdoor Aeroallergens	Control Days (*n* = 170)	Case Days (*n* = 85)	*p*-Value
Alternaria, grains/m^3^	11.85 (16.12)	13.90 (18.39)	0.210
Ambrosia, grains/m^3^	0.09 (0.57)	0.22 (1.43)	0.257
Betula, grains/m^3^	0.19 (1.00)	0.17 (0.89)	0.846
Cupressus, grains/m^3^	7.79 (27.70)	13.93 (80.10)	0.226
Graminaceae, grains/m^3^	6.93 (18.90)	4.97 (11.13)	0.227
Olive, grains/m^3^	1.82 (8.88)	3.67 (14.15)	0.070
Pinaceae, grains/m^3^	11.79 (34.15)	25.95 (96.68)	**0.043**
Urticaceae, grains/m^3^	6.30 (8.76)	6.76 (8.47)	0.618
Total, grains/m^3^	46.77 (63.06)	69.57 (131.31)	**0.017**

**Table 3:** Acute effects of outdoor aeroallergen counts on the risk of admissions overall and by child subgroup: odds ratios (10-grains/m^3^ increase) and 95% confidence intervals through the conditional logistic regression models. Significant effects are in bold.

Lag	Overall (*n* = 85)	Outdoor Sensitization (*n* = 35)	No Outdoor Sensitization (*n* = 47)	Females (*n* = 36)	Males (*n* = 49)	0–4 Years (*n* = 40)	5–17 Years (*n* = 45)
Lag 0	**1.054** **[1.011, 1.098]**	**1.085** **[1.004, 1.173]**	1.037 [0.986, 1.091]	1.034 [0.980, 1.091]	**1.069** **[1.009, 1.132]**	1.030 [0.961, 1.105]	**1.065** **[1.007, 1.127]**
Lag 1	**1.037** **[1.008, 1.067]**	**1.058** **[1.003, 1.115]**	1.025 [0.989, 1.063]	1.026 [0.989, 1.065]	**1.046** **[1.006, 1.088]**	1.018 [0.971, 1.069]	**1.046** **[1.007, 1.087]**
Lag 2	**1.021** **[1.003, 1.039]**	**1.031** **[1.001, 1.061]**	1.014 [0.990, 1.038]	1.018 [0.992, 1.045]	**1.024** **[1.001, 1.048]**	1.007 [0.977, 1.038]	**1.027** **[1.004, 1.052]**
Lag 3	1.005 [0.991, 1.019]	1.004 [0.986, 1.023]	1.002 [0.981, 1.024]	1.01 [0.983, 1.038]	1.003 [0.987, 1.020]	0.995 [0.970, 1.022]	1.009 [0.991, 1.026]
Lag 4	0.989 [0.968, 1.011]	0.979 [0.945, 1.013]	0.991 [0.961, 1.021]	1.002 [0.963, 1.043]	0.982 [0.956, 1.01]	0.984 [0.945, 1.024]	0.991 [0.964, 1.018]
Lag 5	0.974 [0.941, 1.007]	0.954 [0.899, 1.012]	0.979 [0.937, 1.023]	0.994 [0.939, 1.053]	0.962 [0.920, 1.006]	0.973 [0.915, 1.034]	0.973 [0.931, 1.016]

## Data Availability

The data that support the findings of this study are available from the corresponding author upon reasonable request.

## Supplementary Materials

The following supporting information can be downloaded at: https://www.mdpi.com/article/10.3390/ijerph19063586/s1, Figure S1. Power analysis for each aeroallergen based on a range of expected ORs from 1.01 to 1.06. Table S1. Acute effects of outdoor aeroallergen counts on the risk of asthma admissions: odds ratios (10-grains/m^3^ increase) and 95% confidence intervals through the conditional logistic regression models: overall effects (*n* = 85) of each aeroallergen. Table S2. Acute effects of outdoor aeroallergen counts on the risk of asthma admissions overall and by child subgroup: odds ratios (10-grains/m^3^ increase) and 95% confidence intervals through the conditional logistic regression models excluding children below 2 years. Table S3. Acute effects of outdoor aeroallergen counts on the risk of asthma admissions overall and by child subgroup: odds ratios (10-grains/m^3^ increase) and 95% confidence intervals through the conditional logistic regression models excluding cases that occurred before the end of March. Table S4. Acute effects of outdoor aeroallergen counts on the risk of asthma admissions overall and by child subgroup: odds ratios (10-grains/m^3^ increase) and 95% confidence intervals through the conditional logistic regression models: time-stratified design. Table S5. Acute effects of outdoor aeroallergen counts on the risk of asthma admissions overall and by child subgroup: odds ratios (10-grains/m^3^ increase) and 95% confidence intervals through the conditional logistic regression models adjusted by apparent temperature. Table S6. Acute effects of outdoor aeroallergen counts on the risk of asthma admissions overall and by child subgroup: odds ratios (10-grains/m^3^ increase) and 95% confidence intervals through the conditional logistic regression models adjusted by nitrogen dioxide (NO_2_).
