# Supplementary material for: The Effect of Outdoor Aeroallergens on Asthma Hospitalizations in Children in North-Western Tuscany, Italy
Source: Int J Environ Res Public Health. 2022 Mar 17;19(6):3586. doi: 10.3390/ijerph19063586 (PMC8949088; doi:10.3390/ijerph19063586)
Supplement: Supplementary file 1 [file ijerph-19-03586-s001.zip › ijerph-1619295-supplementary.pdf]

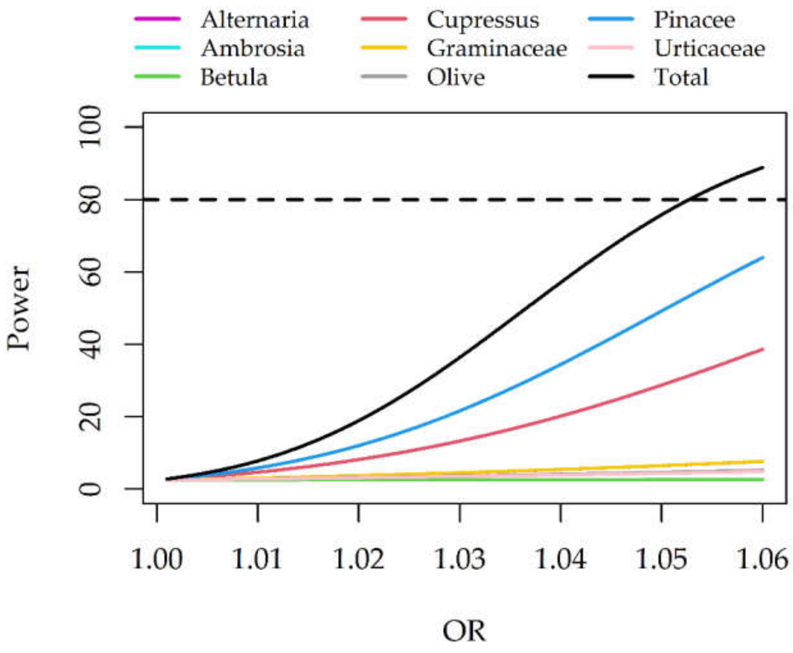

Figure S1. Power analysis for each aeroallergen based on a range of expected ORs from 1.01 to 1.06. Only total aeroallergens reached a power of 80% in the OR range.

Table S1. Acute effects of outdoor aeroallergen counts on the risk of asthma admissions: odds ratios (10-grains/m^3 increase) and 95% confidence intervals through the conditional logistic regression models: overall effects (n=85) of each aeroallergen. Significant effects are in bold.

Lag	Alternaria	Ambrosia	Betula	Cupressus	Graminaceae	Olive	Pinaceae	Urticaceae
Lag 0	**1.140**	4.922	1.473	1.034	0.939	1.751	1.056	1.192
	**[1.020, 1.275]**	[0.2, 121.308]	[0.293, 7.394]	[0.981, 1.090]	[0.791, 1.115]	[0.963, 3.183]	[0.984, 1.134]	[0.959, 1.483]
Lag 1	**1.101**	1.228	1.487	1.015	0.957	1.442	1.041	1.122
	**[1.013, 1.197]**	[0.120, 12.606]	[0.492, 4.493]	[0.980, 1.052]	[0.855, 1.072]	[0.979, 2.123]	[0.991, 1.094]	[0.965, 1.305]
Lag 2	**1.063**	0.306	1.501	0.996	0.976	1.187	1.026	1.056
	**[1.002, 1.128]**	[0.017, 5.640]	[0.503, 4.481]	[0.961, 1.033]	[0.906, 1.052]	[0.985, 1.431]	[0.996, 1.058]	[0.957, 1.166]
Lag 3	1.027	0.076	1.515	0.978	0.995	0.977	1.012	0.994
	[0.979, 1.076]	[0.001, 6.291]	[0.309, 7.416]	[0.927, 1.032]	[0.911, 1.086]	[0.873, 1.095]	[0.991, 1.033]	[0.909, 1.087]
Lag 4	0.991	0.019	1.529	0.960	1.014	0.805	0.997	0.936
	[0.938, 1.047]	[0.001, 9.310]	[0.156, 14.964]	[0.889, 1.037]	[0.883, 1.165]	[0.603, 1.074]	[0.966, 1.029]	[0.819, 1.07]
Lag 5	0.957	0.005	1.544	0.942	1.033	0.663	0.983	0.881
	[0.886, 1.034]	[0.001, 15.192]	[0.074, 32.238]	[0.851, 1.044]	[0.846, 1.262]	[0.403, 1.088]	[0.934, 1.035]	[0.722, 1.074]

Table S2. Acute effects of outdoor aeroallergen counts on the risk of asthma admissions overall and by child subgroup: odds ratios (10-grains/m^3 increase) and 95% confidence intervals through the conditional logistic regression models excluding children below 2 years. Significant effects are in bold.

Lag	Overall (n=75)	Outdoor sensitization (n=35)	No outdoor sensitization (n=38)	Females (n=31)	Males (n=44)	0-4 years (n=30)	5-17 years (n=45)
Lag 0	**1.054**	**1.085**	1.034	1.030	**1.076**	1.031	**1.065**
	**[1.010, 1.099]**	**[1.004, 1.173]**	[0.983, 1.087]	[0.981, 1.082]	**[1.011, 1.146]**	[0.956, 1.112]	**[1.007, 1.127]**
Lag 1	**1.036**	**1.058**	1.021	1.023	**1.050**	1.014	**1.046**
	**[1.007, 1.067]**	**[1.003, 1.115]**	[0.986, 1.058]	[0.989, 1.059]	**[1.007, 1.095]**	[0.962, 1.068]	**[1.007, 1.087]**
Lag 2	**1.019**	**1.031**	1.009	1.017	**1.025**	0.997	**1.027**
	**[1.001, 1.037]**	**[1.001, 1.061]**	[0.985, 1.033]	[0.991, 1.042]	**[1.001, 1.050]**	[0.962, 1.032]	**[1.004, 1.052]**
Lag 3	1.002	1.004	0.997	1.010	1.000	0.980	1.009
	[0.988, 1.017]	[0.986, 1.023]	[0.973, 1.021]	[0.981, 1.039]	[0.983, 1.017]	[0.946, 1.015]	[0.991, 1.026]
Lag 4	0.986	0.979	0.984	1.003	0.975	0.963	0.991
	[0.963, 1.009]	[0.945, 1.013]	[0.950, 1.020]	[0.962, 1.046]	[0.946, 1.005]	[0.913, 1.015]	[0.964, 1.018]
Lag 5	0.969	0.954	0.972	0.997	0.952	0.947	0.973
	[0.935, 1.005]	[0.899, 1.012]	[0.924, 1.023]	[0.940, 1.056]	[0.906, 1.000]	[0.877, 1.022]	[0.931, 1.016]

Table S3. Acute effects of outdoor aeroallergen counts on the risk of asthma admissions overall and by child subgroup: odds ratios (10-grains/m^3 increase) and 95% confidence intervals through the conditional logistic regression models excluding cases that occurred before the end of March. Significant effects are in bold.

Lag	Overall (n=68)	Outdoor sensitization (n=29)	No outdoor sensitization (n=36)	Females (n=26)	Males (n=42)	0-4 years (n=33)	5-17 years (n=35)
Lag 0	**1.064 [1.012, 1.119]**	**1.098 [1.003, 1.201]**	1.047 [0.986, 1.112]	1.050 [0.948, 1.164]	**1.069 [1.007, 1.134]**	1.032 [0.958, 1.112]	**1.087 [1.009, 1.171]**
Lag 1	**1.044 [1.009, 1.081]**	**1.067 [1.004, 1.133]**	1.032 [0.990, 1.075]	1.037 [0.967, 1.113]	**1.047 [1.005, 1.090]**	1.021 [0.970, 1.075]	**1.061 [1.008, 1.116]**
Lag 2	**1.025 [1.004, 1.046]**	**1.037 [1.003, 1.072]**	1.016 [0.989, 1.044]	1.025 [0.982, 1.069]	**1.025 [1.002, 1.050]**	1.010 [0.978, 1.043]	**1.035 [1.005, 1.066]**
Lag 3	1.006 [0.992, 1.021]	1.008 [0.988, 1.027]	1.001 [0.978, 1.025]	1.012 [0.980, 1.046]	1.005 [0.988, 1.021]	0.999 [0.972, 1.026]	1.010 [0.991, 1.028]
Lag 4	0.988 [0.964, 1.012]	0.979 [0.943, 1.017]	0.986 [0.953, 1.021]	1.004 [0.964, 1.046]	0.984 [0.957, 1.012]	0.988 [0.947, 1.030]	0.985 [0.954, 1.017]
Lag 5	0.969 [0.932, 1.008]	0.952 [0.891, 1.016]	0.972 [0.923, 1.023]	0.988 [0.910, 1.072]	0.964 [0.921, 1.009]	0.977 [0.917, 1.042]	0.961 [0.910, 1.015]

Table S4. Acute effects of outdoor aeroallergen counts on the risk of asthma admissions overall and by child subgroup: odds ratios (10-grains/m^3 increase) and 95% confidence intervals through the conditional logistic regression models: time-stratified design. Significant effects are in bold.

Lag	Overall (n=85)	Outdoor sensitization (n=35)	No outdoor sensitization (n=47)	Females (n=36)	Males (n=49)	0-4 years (n=40)	5-17 years (n=45)
Lag 0	**1.046 [1.013, 1.081]**	**1.101 [1.020, 1.188]**	1.024 [0.984, 1.066]	1.027 [0.990, 1.065]	**1.064 [1.016, 1.115]**	1.012 [0.958, 1.069]	**1.069 [1.015, 1.124]**
Lag 1	**1.031 [1.009, 1.055]**	**1.066 [1.013, 1.122]**	1.016 [0.988, 1.045]	1.020 [0.996, 1.045]	**1.043 [1.010, 1.076]**	1.006 [0.968, 1.045]	**1.047 [1.012, 1.084]**
Lag 2	**1.016 [1.003, 1.031]**	**1.033 [1.005, 1.061]**	1.008 [0.989, 1.027]	1.013 [0.996, 1.029]	**1.022 [1.002, 1.041]**	1.000 [0.976, 1.024]	**1.026 [1.006, 1.047]**
Lag 3	1.002 [0.990, 1.014]	1.001 [0.984, 1.018]	0.999 [0.982, 1.017]	1.005 [0.986, 1.026]	1.001 [0.986, 1.016]	0.994 [0.973, 1.015]	1.006 [0.990, 1.021]
Lag 4	0.987 [0.969, 1.007]	0.969 [0.936, 1.003]	0.991 [0.966, 1.017]	0.998 [0.968, 1.030]	0.981 [0.956, 1.006]	0.988 [0.957, 1.020]	0.986 [0.960, 1.012]
Lag 5	0.973 [0.945, 1.002]	0.939 [0.928, 1.001]	0.983 [0.948, 1.020]	0.991 [0.948, 1.037]	0.961 [0.924, 1.001]	0.982 [0.936, 1.030]	0.966 [0.926, 1.007]

Table S5. Acute effects of outdoor aeroallergen counts on the risk of asthma admissions overall and by child subgroup: odds ratios (10-grains/m^3 increase) and 95% confidence intervals through the conditional logistic regression models adjusted by apparent temperature. Significant effects are in bold.

Lag	Overall (n=85)	Outdoor sensitization (n=35)	No outdoor sensitization (n=47)	Females (n=36)	Males (n=49)	0-4 years (n=40)	5-17 years (n=45)
Lag 0	**1.055 [1.011, 1.102]**	**1.090 [1.004, 1.182]**	1.035 [0.984, 1.089]	1.038 [0.976, 1.103]	**1.064 [1.004, 1.128]**	1.031 [0.959, 1.110]	**1.074 [1.010, 1.142]**
Lag 1	**1.039 [1.008, 1.070]**	**1.061 [1.004, 1.121]**	1.025 [0.988, 1.062]	1.030 [0.986, 1.075]	**1.044 [1.003, 1.086]**	1.019 [0.969, 1.072]	**1.052 [1.008, 1.098]**
Lag 2	**1.022 [1.004, 1.041]**	**1.033 [1.002, 1.065]**	1.014 [0.989, 1.040]	1.021 [0.991, 1.052]	**1.024 [1.000, 1.048]**	1.008 [0.976, 1.040]	**1.031 [1.004, 1.058]**
Lag 3	1.006 [0.992, 1.021]	1.006 [0.987, 1.025]	1.004 [0.982, 1.026]	1.013 [0.984, 1.043]	1.005 [0.988, 1.022]	0.996 [0.969, 1.024]	1.010 [0.992, 1.028]
Lag 4	0.99 0 [0.969, 1.013]	0.979 [0.945, 1.015]	0.994 [0.964, 1.024]	1.005 [0.965, 1.047]	0.985 [0.958, 1.013]	0.984 [0.944, 1.027]	0.990 [0.962, 1.018]
Lag 5	0.975 [0.942, 1.009]	0.954 [0.898, 1.013]	0.984 [0.941, 1.028]	0.997 [0.940, 1.058]	0.967 [0.924, 1.011]	0.973 [0.913, 1.037]	0.970 [0.927, 1.015]

Table S6. Acute effects of outdoor aeroallergen counts on the risk of asthma admissions overall and by child subgroup: odds ratios (10-grains/m^3 increase) and 95% confidence intervals through the conditional logistic regression models adjusted by nitrogen dioxide (NO_2). Significant effects are in bold.

Lag	Overall (n=85)	Outdoor sensitization (n=35)	No outdoor sensitization (n=47)	Females (n=36)	Males (n=49)	0-4 years (n=40)	5-17 years (n=45)
Lag 0	**1.054 [1.011, 1.099]**	**1.093 [1.008, 1.185]**	1.037 [0.986, 1.091]	1.034 [0.980, 1.091]	**1.073 [1.011, 1.139]**	1.031 [0.961, 1.106]	**1.067 [1.008, 1.128]**
Lag 1	**1.037 [1.008, 1.068]**	**1.063 [1.006, 1.122]**	1.025 [0.989, 1.063]	1.026 [0.989, 1.065]	**1.049 [1.007, 1.092]**	1.019 [0.971, 1.069]	**1.047 [1.007, 1.088]**
Lag 2	**1.021 [1.003, 1.039]**	**1.033 [1.002, 1.065]**	1.014 [0.990, 1.039]	1.019 [0.992, 1.046]	**1.025 [1.002, 1.050]**	1.007 [0.977, 1.038]	**1.028 [1.004, 1.052]**
Lag 3	1.004 [0.991, 1.019]	1.004 [0.986, 1.023]	1.002 [0.981, 1.024]	1.011 [0.983, 1.040]	1.003 [0.986, 1.019]	0.995 [0.969, 1.022]	1.009 [0.991, 1.026]
Lag 4	0.988 [0.967, 1.010]	0.976 [0.942, 1.012]	0.991 [0.962, 1.022]	1.004 [0.964, 1.046]	0.980 [0.953, 1.008]	0.983 [0.944, 1.024]	0.990 [0.963, 1.018]
Lag 5	0.973 [0.940, 1.007]	0.949 [0.893, 1.009]	0.980 [0.938, 1.024]	0.997 [0.940, 1.056]	0.958 [0.915, 1.003]	0.972 [0.914, 1.033]	0.972 [0.930, 1.016]
